# A preoperative nomogram model for the prediction of lymph node metastasis in buccal mucosa cancer

**DOI:** 10.1002/cam4.6076

**Published:** 2023-05-15

**Authors:** Qian Chen, Rui Wei, Shan Li

**Affiliations:** ^1^ National Clinical Research Center for Geriatric Disorders, Xiangya Hospital Central South University Changsha China; ^2^ Department of Oncology, Xiangya Hospital Central South University Changsha China

**Keywords:** buccal mucosa carcinoma, lymph node metastasis, nomogram

## Abstract

**Objectives:**

We sought to construct a nomogram model predicting lymph node metastasis (LNM) in patients with squamous cell carcinoma of the buccal mucosa based on preoperative clinical characteristics.

**Methods:**

Patients who underwent radical resection of a primary tumor in the buccal mucosa with neck dissection were enrolled. Clinical characteristics independently associated with LNM in multivariate analyses were adopted to build the model. Patients at low risk of LNM were defined by a predicted probability of LNM of less than 5%.

**Results:**

Patients who underwent surgery in an earlier period (January 2015–November 2019) were defined as the model development cohort (*n* = 325), and those who underwent surgery later (November 2019–March 2021) were defined as the validation cohort (*n* = 140). Age, tumor differentiation, tumor thickness, and clinical N stage assessed by computed tomography/magnetic resonance imaging (cN) were independent predictors of LNM. The nomogram model based on these four predictors showed good discrimination accuracy in both the model development and validation cohorts, with areas under the receiver‐operating characteristic curve (AUC) of 0.814 and 0.828, respectively. LNM prediction by the nomogram model was superior to cN in AUC comparisons (0.815 vs. 0.753) and decision curve analysis of the whole cohort. Seventy‐one patients were defined as having a low risk of LNM, among whom the actual metastasis rate was only 1.4%.

**Conclusions:**

A robust nomogram model for preoperative LNM prediction is built.

## 1 INTRODUCTION

Squamous cell carcinoma of the buccal mucosa is a common type of oral cavity cancer.^1^ The incidence of lymph node (LN) metastasis (LNM) at diagnosis is approximately 20%–50%.^2^, ^3^, ^4^, ^5^ Surgery and radiotherapy are the primary definitive treatment modalities for patients without distant metastases.^6^, ^7^ Whether or not there is LNM determines the neck dissection region during surgery and the radiotherapy irradiation field. Misjudgment of LNM before treatment can lead to either overtreatment or insufficient treatment, which can cause unnecessary complications or loco‐regional recurrence. Therefore, it is very important to predict the status of LNM accurately before treatment.

Currently, the most commonly used criteria for determining LNM in clinical practice is the imaging characteristics of the LNs on computed tomography (CT) and/or magnetic resonance imaging (MRI), including their size, shape, clustering, and enhancement pattern.^8^, ^9^ However, the accuracy of this approach is still not satisfactory, with a sensitivity and specificity of 72%–77% and 72%–81%, respectively. Consequently, approximately 20%–30% of patients are misjudged before treatment and thus miss out on the best treatment for them.^10^, ^11^ Positron emission tomography ‐ computed tomography (PET‐CT) has been reported to be able to improve the accuracy of LN prediction,^12^, ^13^, ^14^ but the expensive cost has limited its applications in routine clinical practice. Therefore, identifying more accurate methods with better practicability for predicting LNM before treatment is an urgent interest for clinical oncologists.

It has been reported that several clinical characteristics of cancer patients are associated with LNM, such as age, thickness of the primary tumor, tumor differentiation, and T stage.^15^, ^16^, ^17^, ^18^, ^19^ Combining these factors with CT/MRI characteristics would be a potential way to improve the accuracy of predicting LNM. A nomogram is a graphic calculation tool for predicting the probability of a clinical event based on the combination of several predictors. The nomogram model has become a widely used tool in the diagnosis and treatment of head and neck cancers, such as predicting the risk of loco‐regional recurrences,^20^ distant metastases,^21^ treatment‐related benefits,^22^ and treatment‐related complications.^23^ Previous studies have developed nomogram models to estimate the risk of LNM in several head and neck cancers, including papillary thyroid carcinoma,^24^ laryngeal squamous cell carcinoma,^25^ and hypopharyngeal cancer.^26^ However, such models have not been reported in squamous cell carcinoma of the buccal mucosa. Therefore, we performed the current study to develop a nomogram model capable of predicting LNM preoperatively.

## 2 METHODS

### 2.1 Patient selection

Patients were enrolled according to the following criteria: (1) pathologically confirmed squamous carcinoma of the buccal mucosa, (2) having undergone radical resection of the primary tumor with neck dissection at our hospital between January 2015 and March 2021, (3) CT/MRI records available for review, (4) no prior systemic treatment or radiotherapy before surgery, and (5) no other coexisting malignancies. The current study was approved by the ethics committee of our institute and written informed consent for study inclusion was obtained from each participant.

### 2.2 Univariate and multivariate analyses of LNM

The final status of LNM was determined by the postoperative pathological stage of LNs (pN). For patients who were judged as lymph nodes‐negative before surgery, selective neck dissections (END) involving levels I–III were performed. For patients who were judged as lymph nodes‐positive before surgery, comprehensive neck dissections involving levels I–V were performed.^27^ To investigate the association between clinical characteristics and pN, univariate and multivariate analyses were conducted in the model development cohort. Clinical characteristics reported to be associated with LNM were included in the univariate analysis, including sex, age, clinical T stage (cT), thickness of the primary tumor, differentiation grade, and clinical N stage assessed by pretreatment CT/MRI (cN). The T stage and N stage were categorized according to the seventh American Joint Committee on Cancer (AJCC) staging system. The tumor thickness was measured as previously described.^28^ The cutoff value for age and tumor thickness was determined by the receiver‐operating characteristic curve (ROC) analysis and maximal Youden's index. The positive status of cN was defined by the characteristics of CT/MRI imaging, including LNs measuring greater than 10 mm along the shortest diameter; three or more contiguous and confluent LNs, each with a shortest diameter of 8–10 mm; LNs of any size with central necrosis or a contrast‐enhanced rim; and LNs of any size with extracapsular extension.^8^, ^9^ The multivariate analysis included the variates demonstrating *α* < 0.1 in the univariate analysis.

### 2.3 Construction and validation of the nomogram model

Seventy percent of the enrolled patients, who underwent surgery in an earlier period, were defined as the model development cohort, and the other 30% of patients, who underwent surgery in a later period, were defined as the validation cohort. The model development cohort was used for model construction and internal validation, while the validation cohort was used for external validation of the model. The nomogram model was constructed using the “rms” package in R version 4.1.0 (R Foundation for Statistical Computing), with the predictors identified by multivariate analysis. The calibration plot was performed via a bootstrap method with 1000 resamples, with the parameters of “method = boot” and “B = 1,000.” The ROC curve was plotted, and the area under the ROC curve (AUC) was adopted to estimate the accuracy of the nomogram model in predicting LNM, accompanied by the Hosmer–Lemeshow test.

### 2.4 Comparisons of the nomogram model and cN for predicting LNM

To investigate whether the nomogram model improved the accuracy of predicting LNM relative to cN, which is the routine method in clinical practice, the values of AUC of these two methods were compared in the model development cohort, validation cohort, and the whole study cohort. A decision curve analysis was performed in the whole cohort using the “rmda” package in R to evaluate the clinical value of the nomogram model and cN by calculating the net benefits when different threshold probabilities were set.^29^

### 2.5 Statistical analyses

The Statistical Package for the Social Sciences version 25 (IBM Corporation) and R software programs were used to conduct the statistical analyses. Comparisons of baseline characteristics between the model development cohort and the validation cohort were performed with the chi‐squared test. Univariate and multivariate analyses of LNM were completed with the logistic regression model. Patients with a predicted probability of LNM of less than 5% were defined as the low‐risk group.

## 3 RESULTS

### 3.1 Patients and clinical characteristics

**FIGURE 1:**
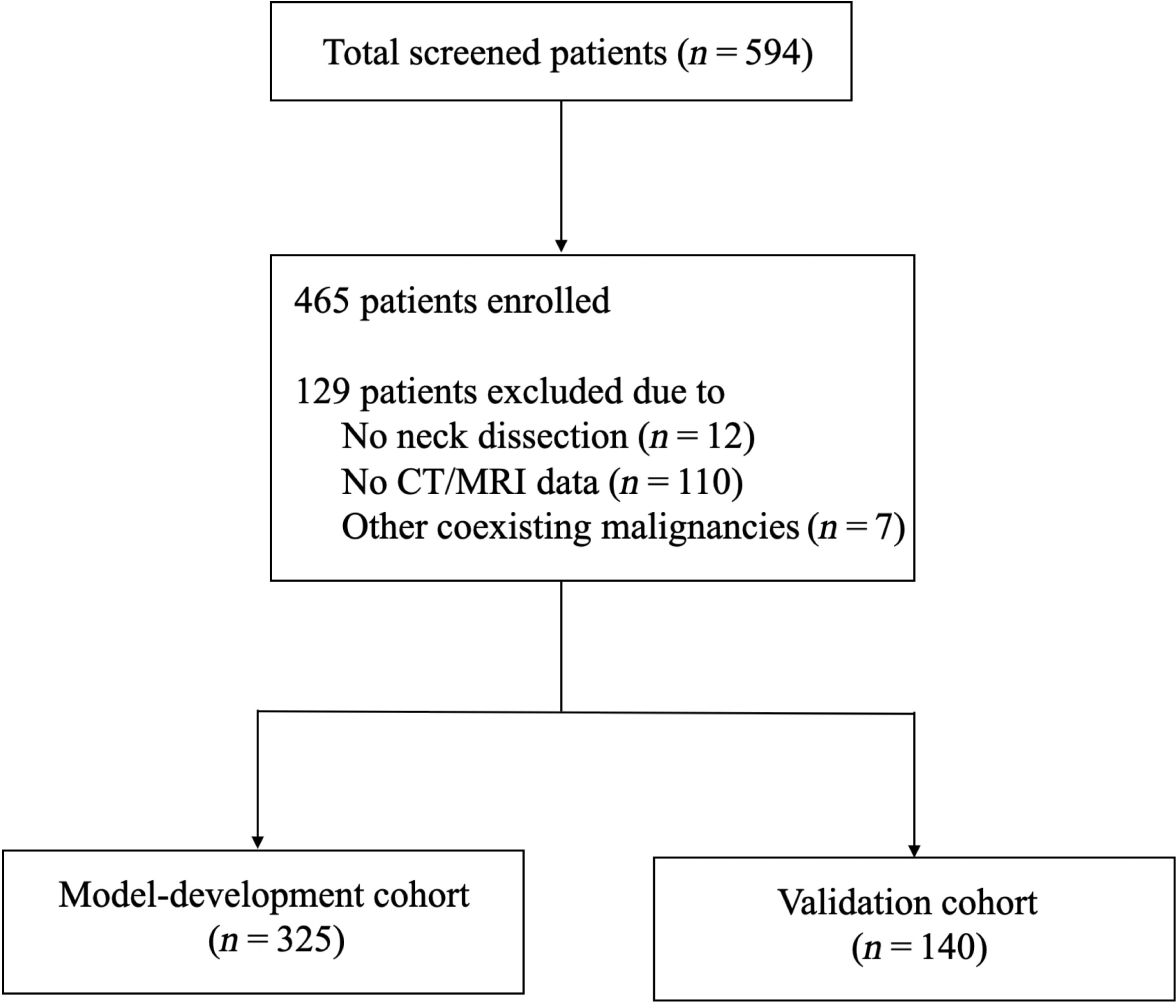
Flowchart of patient enrollment.

As shown in Figure 1, a total of 594 patients with squamous cell carcinoma of the buccal mucosa were screened, and 465 patients were finally enrolled. Patients who underwent surgery in an earlier period (January 2015–November 2019) were defined as the model development cohort (*n* = 325), and those who underwent surgery later (November 2019–March 2021) were defined as the validation cohort (*n* = 140). Table 1 shows the clinical characteristics of the model development and validation cohorts. The cutoff value for age (cutoff value = 60 years) and tumor thickness (cutoff value = 9.5 mm) were determined by ROC curve analysis. There was no significant difference in these clinical characteristics between the model development and validation cohorts.

**TABLE 1:** Clinical characteristics of patients in the model development and validation cohorts.

	Model development cohort	Validation cohort	*p*
	(*n* = 325)	(*n* = 140)	
Race			
Asian	325	140	
Gender			0.066
Female	21	3	
Male	304	137	
Age			0.470
≤60 years	276	123	
>60 years	49	17	
T stage			0.732
T1–2	240	101	
T3–4	85	39	
Tumor thickness			0.738
≤9.5 mm	92	41	
>9.5 mm	233	99	
Tumor differentiation			0.830
Moderate/low	118	48	
Well	207	92	
cN^a^			0.225
Negative	160	78	
Positive	165	62	
LNM			0.387
No	225	91	
Yes	100	49	

Abbreviations: cN, clinical N stage; LNM, lymph node metastasis.

^a^ cN was assessed by characteristics of LNs on CT and/or MRI.

### 3.2 Construction of nomogram model

**FIGURE 2:**
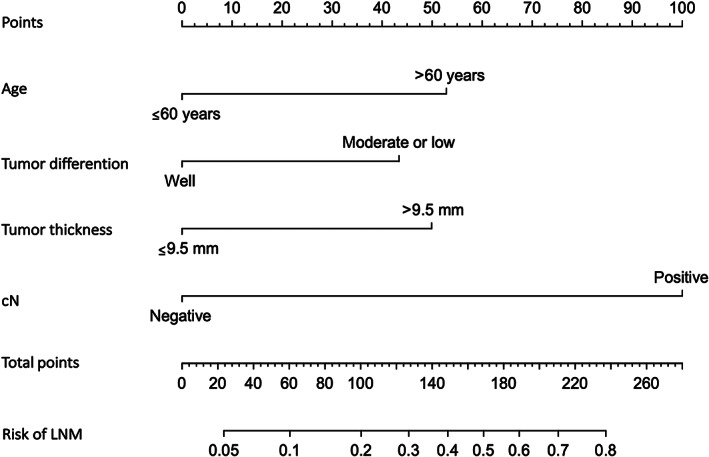
The nomogram predicting the risk of LNM in patients with squamous cell carcinoma of the buccal mucosa. The total points of each patient were calculated by drawing an upward vertical line to “Points,” and the corresponding risk of LNM was calculated by drawing a downward vertical line from “Total Points.” cN, clinical N stage; LNM, lymph node metastasis.

As shown in Table 2, age (≤60 years vs. >60 years; odds ratio [OR], 2.928; 95% confidence interval [CI], 1.417–6.049; *p* = 0.004), tumor differentiation (moderate/poor vs. well; OR, 0.409; 95% CI, 0.233–0.718; *p* = 0.002), tumor thickness (≤9.5 mm vs. >9.5 mm; OR, 2.488; 95% CI, 1.109–5.580; *p* = 0.027), and cN (negative vs. positive; OR, 7.348; 95% CI, 3.876–13.930; *p* < 0.001) were independent factors associated with LNM. Figure 2 shows the nomogram model constructed based on these four predictors. The total points of each patient were calculated by drawing an upward vertical line to “Points,” and its corresponding risk of LNM was calculated by drawing a downward vertical line from “Total Points.”

**TABLE 2:** Univariate and multivariate logistic regression analyses of LNM.

	Univariate analysis			Multivariate analysis		
	OR	95% CI	*p*	OR	95% CI	*p*
Gender			0.454			‐
Female	1.000	reference		‐	‐	
Male	0.705	0.283,1.759		‐	‐	
Age			0.003			0.004
≤60 years	1.000	reference		1.000	reference	
>60 years	2.526	1.369,4.693		2.928	1.417,6.049	
T stage			0.001			0.155
T1–2	1.000	reference		1.000	reference	
T3–4	2.321	1.385,3.889		1.544	0.848,2.809	
Tumor thickness			<0.001			0.027
≤9.5 mm	1.000	reference		1.000	reference	
>9.5 mm	5.161	2.544,10.471		2.488	1.109,5.580	
Tumor differentiation			0.004			0.002
Moderate/low	1.000	reference		1.000	reference	
Well	0.383	0.236,0.623		0.409	0.233,0.718	
cN^a^			<0.001			<0.001
Negative	1.000	reference		1.000	reference	
Positive	9.333	5.123,17.005		7.348	3.876,13.930	

Abbreviations: cN, clinical N stage; LNM, lymph node metastasis; OR, odds ratio.

^a^ cN was assessed by characteristics of LNs on CT and/or MRI.

### 3.3 Accuracy of the nomogram model

**FIGURE 3:**
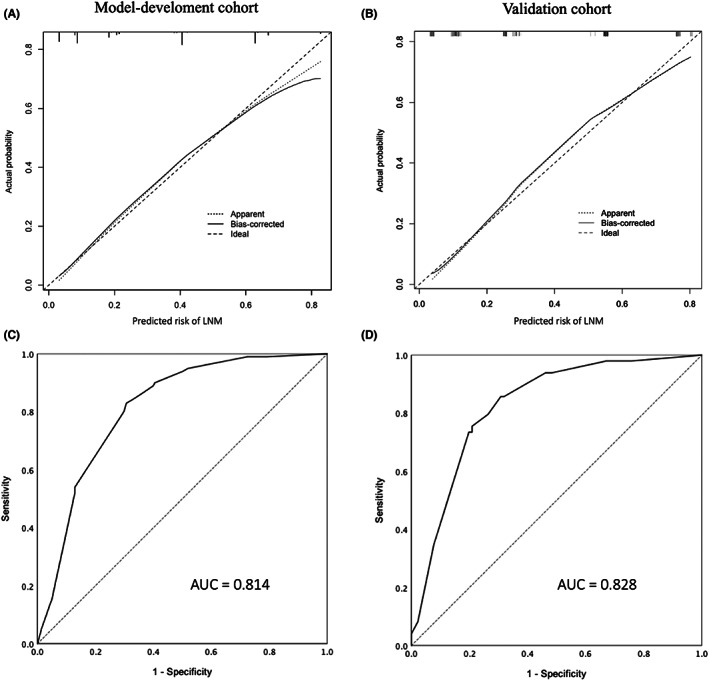
Internal and external validation of the nomogram model. (A) Calibration plot of the nomogram model for the model development cohort. (B) Calibration plot of the nomogram model for the validation cohort. (C) The ROC curve of the model development cohort. The AUC was 0.814 (95% CI, 0.767–0.861; Hosmer–Lemeshow test, *p* = 0.538). (D) The ROC curve of the validation cohort. The AUC was of 0.828 (95% CI, 0.759–0.898; Hosmer–Lemeshow test, *p* = 0.838).

The accuracy of the nomogram model in predicting LNM was validated both internally and externally in the model development and validation cohorts, respectively. For internal validation, the calibration plot showed that the risk predicted by the nomogram model was close to the actual probability of LNM (Figure 3A). In addition, the nomogram model exhibited good discrimination accuracy, with an AUC of 0.814 (95% CI, 0.767–0.861; Hosmer–Lemeshow test, *p* = 0.538) for the prediction of LNM (Figure 3C). During external validation, the nomogram model showed both a similar prediction accuracy in the calibration plot (Figure 3B) and discrimination accuracy, with an AUC of 0.828 (95% CI, 0.759–0.898; Hosmer–Lemeshow test, *p* = 0.838) (Figure 3D).

### 3.4 Comparisons of the nomogram model and cN for predicting LNM

**FIGURE 4:**
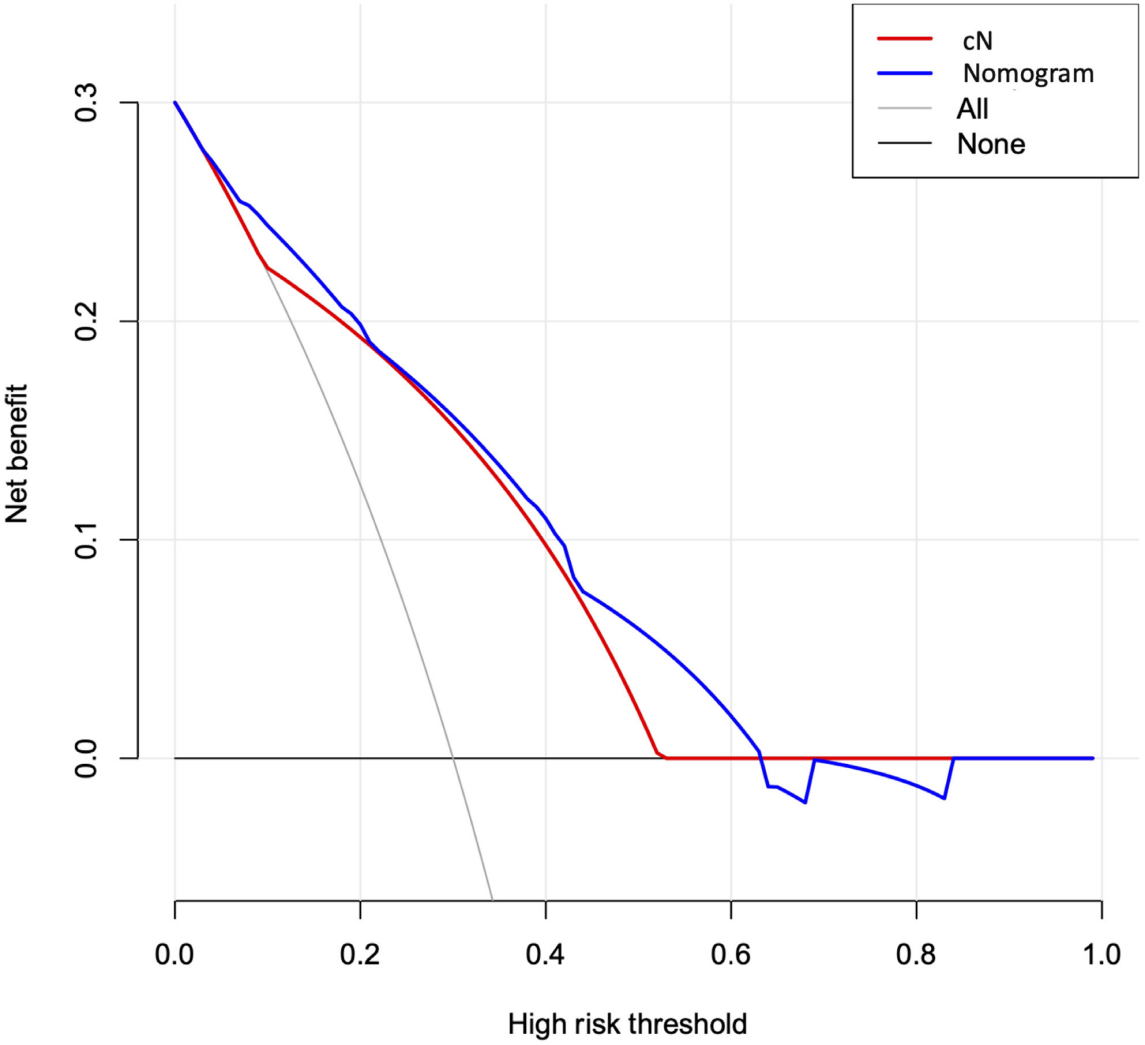
Decision curve analysis for the cN approach and the nomogram model. The x‐axis and y‐axis represent the threshold probability and net benefit, respectively. “All” and “None” refer to the assumptions that all patients and no patients have LNM, respectively.

As shown in Table 3, the nomogram model improved the accuracy of predicting LNM over that of cN in the model development cohort (AUC, 0.814 vs. 0.740), the validation cohort (AUC, 0.828 vs. 0.787), and the whole cohort (AUC, 0.815 vs. 0.753). In addition, the decision curve analysis indicated that, when the threshold probability is within the range of 0.04–0.63, using the nomogram model to predict LNM adds more net benefit than using cN (Figure 4).

**TABLE 3:** The AUC comparison of the nomogram model and cN for predicting LNM.

	Nomogram model	cN^a^
Model developmentcohort	0.814	0.740
Validation cohort	0.828	0.787
Whole cohort	0.815	0.753

Abbreviations: AUC, area under the receiver‐operating characteristic curve; LNM, lymph node metastasis; cN, clinical N stage.

^a^ cN was assessed by characteristics of LNs on CT and/or MRI.

### 3.5 Identification of patients at low risk of LNM

To identify the patients at low risk of LNM and avoid providing unnecessary treatment to these individuals, those with a predicted probability of LNM of less than 5% were defined as the low‐risk group. Ultimately, the nomogram model identified 71 out of 465 patients (15.3%) in the whole cohort as being at low risk of LNM. In the low‐risk group, the actual metastasis rate was only 1.4% (1/71).

## 4 DISCUSSION

Accurate prediction of LNM before treatment is critical for determining the optimal treatment plan of surgery and radiotherapy for patients with squamous cell carcinoma of the buccal mucosa. The routine method of LNM prediction in clinical practice, which is based on the imaging characteristics of LNs, is not satisfactory.^10^, ^11^ In our study, 27.5% of patients were misjudged before surgery by cN, which is consistent with findings of previous reports.^10^, ^11^ How to improve the accuracy of LNM prediction with simple and practical predictors is an urgent concern for clinical oncologists. To the best of our knowledge, the current study is the first to investigate the application of a nomogram model based on patient clinical characteristics for predicting LNM in squamous cell carcinoma of the buccal mucosa.

The multivariate analysis of our study showed that age (≤60 years vs. >60 years), tumor thickness (≤9.5 mm vs. >9.5 mm), tumor differentiation (moderate/poor vs. well), and cN were independent predictors of LNM. To facilitate the construction of the nomogram model, the grade of tumor differentiation was divided into two categories (moderate/poor and well). Similarly, age and tumor thickness were transformed into dichotomous variables, with the cutoff value determined by ROC curves. Our multivariate analysis results were in concordance with previous studies, which also reported that LNM was associated with age,^30^ tumor thickness,^31^, ^32^, ^33^ tumor differentiation,^32^, ^34^ and cN^10^, ^11^ in buccal mucosa carcinoma.

Our nomogram model based on these four predictors showed good accuracy of prediction and discrimination in the model development cohort (Figure 3A,C). The accuracy of the nomogram model was further confirmed in the validation cohort (Figure 3B,D). Moreover, compared with cN, the nomogram model was superior in predicting LNM, as it exhibited advantages in the comparisons of AUC (Table 3) and added more net benefit in the decision curve analysis (Figure 4). Besides, the performance of our nomogram is comparable to PET‐CT which has been reported to have an AUC of 0.804–0.819 in the LN prediction of oral cavity squamous cell carcinoma in previous studies.^13^, ^14^ In addition, the accuracy of our nomogram model is also comparable to that documented for robust nomogram models designed for predicting LNM in other types of cancers, which demonstrated AUCs of 0.789 to 0.878.^29^, ^35^, ^36^, ^37^

It is worth mentioning that lymphovascular invasion (LVI), perineural invasion (PNI), and depth of infiltration (DOI) of the primary tumor, which have been reported to be convincing predictors of LNM in previous studies,^38^, ^39^, ^40^ were not included in the current study. For preoperative biopsy samples, these pathological parameters could not be assessed accurately in most cases due to the small volume of specimens. Recent studies have indicated that preoperative radiological DOI (rDOI) assessed by contrast‐enhanced CT or multiparametric MRI showed good consistency with pathological DOI (pDOI) and could be a potential replacement for pDOI in oral cavity cancer.^41^, ^42^, ^43^ Similarly, preoperative evaluation of LVI and PNI by radiology (rLVI and rPNI) has also been reported to be feasible in head and neck cancers.^44^, ^45^ Incorporating rDOI, rLVI, and rPNI into the nomogram model may further improve the accuracy of LNM prediction in future study.

For patients at low risk of LNM, reducing the extent of neck dissection and the radiotherapy field is under investigation to minimize treatment‐related complications and improve their quality of life without decreasing survival rates.^46^ With the proposed nomogram model, the risk of LNM can be quantified, and we defined patients with a predicted probability of LNM of less than 5% as the low‐risk group in the current study; ultimately, in the low‐risk group identified by our nomogram model, the actual metastasis rate was only 1.4% (1/71). However, for patients who were predicted to be without LNM by cN, the actual metastasis rate was 10.5%, which is similar to findings of previous reports.^10^ Therefore, our nomogram model has an obvious advantage in identifying patients at low risk of LNM.

Occult LNM in patients with cN0 is an important reason for insufficient treatment and tumor recurrence in oral cavity cancer.^47^, ^48^ How to detect occult LNM in patients with cN0 before treatment is a critical issue for physicians. With our nomogram model, the risk of occult LNM can be estimated quantitatively. For a cN0 patient who has none, one, two, or three of the other three risk factors in the model (age > 60 years, tumor thickness > 9.5 mm, and moderate/poor differentiation), the risk of occult LNM is estimated to be around <5%, 5%–10%, 20%, or 40%, respectively. Therefore, our model can serve as a clinical decision‐making tool to provide references for surgeons and radiation oncologists to decide the extent of neck dissection and irradiation.

It should be mentioned that the seventh edition AJCC staging system was adopted due to the lack of DOI information in a large proportion of patients who received treatment before 2018, which is a major limitation of our study. Compared with the seventh edition, the eighth edition incorporated DOI and extranodal extension to modify the TNM stage. It has been reported that approximately 10%–25.8% of patients with oral squamous cell carcinoma (OSCC) were upstaged when switching from the seventh to the eighth edition.^49^, ^50^ Of note, the T stage was not an independent factor associated with LNM in our study (Table 2). As the eighth edition incorporated the information of DOI, which has been regarded as a relevant factor of LNM,^38^, ^39^, ^40^ the association between the T stage and LNM may be changed if the eighth edition was used.^51^ Besides, the eighth edition has shown better accuracy in predicting the survival outcomes of OSCC patients and is more commonly recommended in clinical practice.^49^, ^50^, ^52^ Taken together, the eighth edition would be a more desirable choice in future studies, and the issue of the AJCC edition should be noted while interpreting the results of the current study.

The results of the current study were potentially affected by several factors. First, all of the included patients were from a single center. Although our model was validated using an independent external cohort, it needs to be further validated by other institutions. Second, 110 patients were excluded during patient enrollment due to a lack of CT/MRI data, which may have led to selection basis. Third, the sensitivity of the nomogram model for identifying patients at low risk of LNM is relatively low. Among 316 patients without LNM, only 70 patients were identified as being at low risk of LNM by the model.

To summarize, a robust nomogram model based on four common clinical characteristics is built for LNM prediction. The nomogram can be used as a clinical decision‐making tool in helping determine the extent of neck dissection and irradiation for surgeons and radiation oncologists.

## AUTHOR CONTRIBUTIONS

**Qian Chen:** Data curation (lead); formal analysis (lead); methodology (lead); writing – original draft (lead). **Rui Wei:** Conceptualization (supporting); funding acquisition (lead); supervision (supporting); writing – review and editing (supporting). **Shan Li:** Conceptualization (lead); supervision (lead); validation (lead); writing – original draft (supporting); writing – review and editing (lead).

## ETHICS APPROVAL STATEMENT

This study was approved by the Ethics Committee of the Xiangya Hospital of Central South University prior to commencement (approval number: 202011169).

## Data Availability

The data that support the findings of this study are available on request from the corresponding author. The data are not publicly available due to privacy or ethical restrictions.
